# Clinical implications of the blood urea nitrogen/creatinine ratio in heart failure and their association with haemoconcentration

**DOI:** 10.1002/ehf2.12531

**Published:** 2019-12-09

**Authors:** Yasumori Sujino, Shintaro Nakano, Jun Tanno, Yasuyuki Shiraishi, Ayumi Goda, Atsushi Mizuno, Yuji Nagatomo, Takashi Kohno, Toshihiro Muramatsu, Shigeyuki Nishimura, Shun Kohsaka, Tsutomu Yoshikawa, for the West Tokyo Heart Failure Registry Investigators

**Affiliations:** ^1^ Department of Cardiology Saitama Medical University, International Medical Center Saitama Japan; ^2^ Department of Cardiology Keio University School of Medicine Tokyo Japan; ^3^ Division of Cardiology Kyorin University School of Medicine Tokyo Japan; ^4^ Department of Cardiology St. Luke's International Hospital Tokyo Japan; ^5^ Department of Cardiology National Defense Medical College Saitama Japan; ^6^ Department of Cardiology Sakakibara Heart Institute Tokyo Japan

**Keywords:** Acute decompensated heart failure, Blood urea nitrogen/creatinine ratio, Haemoconcentration, Haemodilution

## Abstract

**Aims:**

The blood urea nitrogen (BUN)/creatinine ratio is a strong prognostic indicator in patients with acute decompensated heart failure (ADHF). However, the clinical impact of a high BUN/creatinine ratio at discharge with respect to renal dysfunction, neurohormonal hyperactivity, and different responsiveness to decongestion therapy remains unclear. Herein, we examined (i) the predictive value of a high BUN/creatinine ratio at discharge and (ii) its haemoconcentration‐dependent effects, in patients with ADHF.

**Methods and results:**

The West Tokyo Heart Failure registry was a multicentre, prospective cohort registry‐based study that enrolled patients hospitalized with a diagnosis of ADHF. The endpoint was post‐discharge all‐cause death. Based on the degree of haemoconcentration, patients (*n* = 2090) were divided into four subcategories. In multivariate proportional hazard analyses, a higher BUN/creatinine ratio was independently associated with higher all‐cause mortality in the total population and in the extreme haemodilution (ΔHaemoglobin ≤ −0.9 g/dL) and haemoconcentration (0.8 g/dL ≤ ΔHaemoglobin) subcategories, but not in the modest haemodilution/haemoconcentration subcategories.

**Conclusions:**

A higher BUN/creatinine ratio at discharge was independently associated with higher post‐discharge all‐cause mortality in patients with ADHF. The predictive value of a high BUN/creatinine ratio at discharge was haemoconcentration dependent and may be an unfavourable predictor in patients showing excessive haemoconcentration and haemodilution, but not in those showing modest haemoconcentration/haemodilution.

## Introduction

Renal dysfunction is a common co‐morbidity in patients with acute decompensated heart failure (ADHF).^1^, ^2^ A higher blood urea nitrogen (BUN)/creatinine ratio is considered to be associated with mortality in patients with HF and co‐morbid renal dysfunction.^1^ Although the normal values are unclear,^3^ the BUN/creatinine ratio is used clinically as a marker for ‘prerenal’ renal dysfunction and as a metric of sympathetic and neurohormonal hyperactivity.^1^, ^4^, ^5^ Previous studies have investigated the clinical significance of the BUN/creatinine ratio collected at hospital admission.^2^, ^3^, ^4^ However, the clinical significance of a high BUN/creatinine ratio at hospital discharge following in‐hospital decongestion therapy is unknown. A high BUN/creatinine ratio at discharge may result from inappropriate in‐hospital decongestion therapy. For example, haemoconcentration is conventionally used as a marker for decongestion therapy, although a practical haemoconcentration target to achieve proper fluid removal while avoiding unfavourable renal dysfunction remains to be defined.^6^

Herein, we hypothesized that a high BUN/creatinine ratio at discharge is an integrated marker composed of renal and prerenal dysfunction, neurohormonal hyperactivity, and various degrees of responsiveness to in‐hospital decongestion therapy. Further, we hypothesized that the clinical implications of a high BUN/creatinine ratio at discharge may differ according to the response to decongestion therapy, as indicated by the degree of haemoconcentration (see schematic illustration of our hypothesis in Supporting Information, *Figure* *S1*). Thus, a high BUN/creatinine ratio may be unfavourable if haemodilution or haemoconcentration is excessive, whereas it may be less meaningful if haemodilution or haemoconcentration is mild. The aim of this study was to examine (i) the predictive value of a high BUN/creatinine ratio at discharge and (ii) its specific effects dependent on the degree of haemoconcentration, in patients with ADHF.

## Methods

### Study design

The design of the West Tokyo Heart Failure (WET‐HF) registry was previously reported.^7^ Briefly, the WET‐HF registry was a multicentre, prospective cohort registry‐based study that enrolled all patients hospitalized with a diagnosis of ADHF according to the Framingham acute HF criteria. Patients presenting with acute coronary syndrome were not included. The five study centres involved in the registry were located in Tokyo and Saitama in Japan and included three university hospitals (Keio University, Kyorin University, and Saitama Medical University International Medical Center) and two tertiary referral hospitals (Sakakibara Heart Institute and St. Luke's International Hospital). Before the launch of the WET‐HF registry, information on the objective and social significance of the present study, as well as an abstract, were provided for clinical trial registration with the University Hospital Medical Information Network (UMIN000001171). Informed consent was obtained from each patient before the study. Nearly all of the enrolled patients were Japanese. The present study was approved by the ethics review committee of each centre.

### Eligibility criteria

**Figure 1:**
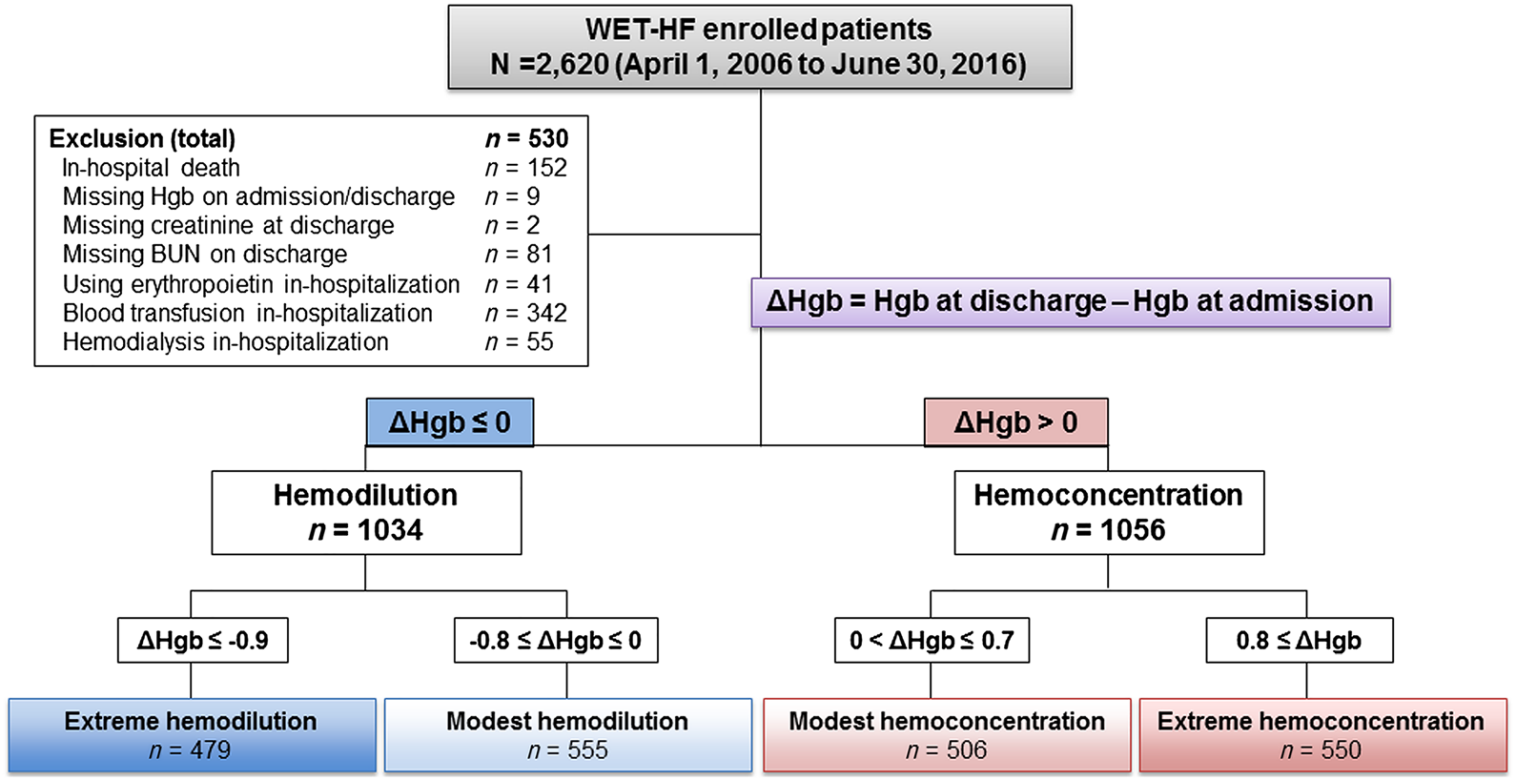
Eligibility criteria and classification according to change in haemoglobin concentration during hospitalization. BUN, blood urea nitrogen; Hgb, haemoglobin; WET‐HF, West Tokyo Heart Failure registry.

Among all patients enrolled from April 2006 to June 2016, we analysed the data for 2620 patients who survived to discharge and who were followed up. Within our cohort, 530 patients were excluded because of death during hospitalization (*n* = 152, 5.08%), missing serum haemoglobin data at admission and discharge (*n* = 9, 0.34%), missing serum creatinine (*n* = 2, 0.07%) and BUN (*n* = 81, 3.09%) data at discharge, the use of erythropoietin (*n* = 41, 1.56%) and blood transfusion during hospitalization (*n* = 342, 13.05%), and haemodialysis performed previously and/or during hospitalization (*n* = 55, 2.09%). The remaining 2090 patients were included in the present analysis (*Figure* *1*).

### Data collection and definitions

Laboratory findings including haemoglobin, serum sodium, creatinine, and BUN levels were recorded at admission and discharge. Haemoconcentration was defined as an increased haemoglobin level from admission to discharge.^8^ The change in the haemoglobin concentration from admission to discharge (ΔHgb) was also calculated. These data were analysed in the total population and in each of the four ΔHgb‐based subcategories divided by the quartiles of ΔHgb as follows: (i) extreme haemodilution (ΔHgb ≤ −0.9 g/dL), (ii) modest haemodilution (−0.8 g/dL ≤ ΔHgb ≤ 0.0 g/dL), (iii) modest haemoconcentration (0.0 g/dL < ΔHgb ≤ 0.7 g/dL), and (iv) extreme haemoconcentration (0.8 g/dL ≤ ΔHgb).

Vital signs including heart rate and systolic blood pressure at discharge were recorded. Worsening renal function during hospitalization was defined as an increase in the serum creatinine level of ≥0.3 mg/dL from admission to discharge.^9^, ^10^ Chronic kidney disease was defined as an estimated glomerular filtration rate of <60 mL/min/1.73 m^2^.

### Endpoint

The study endpoint was post‐discharge all‐cause death. The median follow‐up period was 771 days (interquartile range, 400–1086 days), and the total follow‐up time was 4630.7 person‐years.

### Statistical analysis

Data are presented as mean ± standard deviation or median (first–third quartile) for continuous variables and as number (proportion) for categorical variables. The Shapiro–Wilk test was performed for normality. Inter‐subcategorical comparisons were performed using analysis of variance or Wilcoxon's rank sum test for continuous variables, and the chi‐square test was used for categorical variables. Univariate Cox regression analyses were performed to investigate the association of independent variables with post‐discharge all‐cause death. Multivariate Cox proportional regression analyses were subsequently performed to identify potential predictors of all‐cause death. In the multivariate analysis, in addition to the BUN/creatinine ratio at discharge, established predictive factors for ADHF including age, left ventricular ejection fraction (LVEF), and treatment with angiotensin‐converting enzymes (ACE‐I) or angiotensin receptor blockers (ARB), loop diuretics, and beta‐blockers at discharge^11^, ^12^, ^13^, ^14^, ^15^, ^16^ were initially included in the model (Model 1). Next, the independent variables that were significantly associated with all‐cause mortality in the univariate analyses, including ischaemic aetiology, systolic blood pressure, and resting heart rate, were included in the model, in addition to the aforementioned established predictive factors (Model 2). Kaplan–Meier analyses with a log‐rank test were performed to evaluate the predictive value of a high BUN/creatinine ratio at discharge for post‐discharge all‐cause mortality using the median value (22.1) as a cut‐off point. A value of *P* < 0.05 was considered significant for individual comparisons, and Bonferroni correction for multiple comparisons was performed as necessary. All statistical analyses were performed using statistical software (JMP® 10; SAS Institute Inc., Cary, NC, USA).

## Results

### Patient characteristics

The baseline characteristics and variables at admission and discharge in all 2090 patients and in the four ΔHgb‐based subcategories are shown in *Table* 1 (for abstracted representative variables) and Supporting Information, *Table S1*(*A*) and (*B*) (for all analysed variables). Among the baseline characteristics, age and the proportion of patients with ischaemic aetiology were significantly different between the subcategories; patients were relatively older in the modest haemodilution and haemoconcentration ΔHgb‐based subcategories, and the proportion of patients with an ischaemic aetiology was higher in the extreme and modest haemodilution subcategories. The proportion of chronic kidney disease was 63.0% and was similar between the four ΔHgb‐based subcategories.

**Table 1:** Baseline characteristics, laboratory findings at admission, and variables at discharge in the total population and four haemoglobin concentration level groups from admission to discharge (ΔHgb)‐based subcategories

	Total (*n* = 2090)	Extreme haemodilution (*n* = 479)	Modest haemodilution (*n* = 555)	Modest haemoconcentration (*n* = 506)	Extreme haemoconcentration (*n* = 550)	*P* value (between groups)
Baseline characteristics						
Age (years)† ^,^ ‡ ^,^ || ^,^ #	76.0 (65.0–83.0)	75.0 (63.0–83.0)	76.0 (67.0–84.0)	77.0 (67.0–83.0)	75.0 (63.0–82.0)	0.0020*
Men	1294 (61.9)	309 (64.5)	337 (60.7)	308 (60.8)	340 (61.8)	0.5828
CKD	1318 (63.0)	324 (67.6)	357 (64.3)	309 (61.0)	328 (59.6)	0.0389
Ischaemic aetiology‡ ^,^ § ^,^ ||	594 (28.4)	161 (33.6)	173 (31.1)	117 (23.1)	143 (26.0)	0.0007*
Laboratory findings at admission						
Haemoglobin (g/dL)† ^,^ ‡ ^,^ § ^,^ ||	12.4 (10.9–13.9)	13.4 (12.1–14.9)	12.3 (10.9–13.7)	12.2 (10.6–13.5)	11.8 (10.4–13.4)	<0.0001*
Creatinine (mg/dL)	1.00 (0.77–1.30)	1.03 (0.80–1.34)	1.00 (0.80–1.33)	0.96 (0.76–1.30)	0.94 (0.73–1.24)	0.0040*
BUN (mg/dL)§	20.8 (16.0–28.3)	21.9 (16.6–30.2)	21.7 (16.4–29.0)	20.8 (16.1–27.6)	19.3 (14.7–26.5)	0.0001*
BUN/creatinine ratio	20.7 (16.8–26.1)	20.6 (16.3–27.0)	21.0 (17.1–25.8)	20.7 (17.0–25.8)	20.3 (16.6–26.2)	0.5556
Vital signs, echocardiographic and laboratory findings, medications at discharge						
Systolic BP (mmHg)	110 (100–122)	110 (100–124)	110 (98–122)	110 (100–122)	110 (100–120)	0.4218
Resting HR (beats/min)	70 (61–78)	70 (61–80)	70 (60–78)	70 (62–78)	70 (61–80)	0.4239
WRF (ΔCr > 0.3)	211 (10.0)	55 (11.4)	64 (11.5)	43 (8.5)	49 (8.9)	0.2063
LVEF (%)	44.0 (31.0–58.0)	43.0 (30.0–56.0)	42.0 (30.0–58.0)	45.0 (32.0–58.0)	45.0 (32.0–59.0)	0.0440
Laboratory findings						
Haemoglobin (g/dL)† ^,^ ‡ ^,^ § ^,^ || ^,^ # ^,^ **	12.4 (10.9–13.9)	11.6 (10.5–12.9)	12.0 (10.7–13.4)	12.5 (11.0–14.0)	13.3 (11.9–15.0)	<0.0001*
Creatinine (mg/dL)	1.10 (0.80–1.30)	1.00 (0.76–1.91)	1.01 (0.80–1.40)	1.00 (0.80–1.30)	0.99 (1.80–1.25)	0.1735
BUN (mg/dL)	22.4 (16.7–31.0)	21.0 (16.0–29.6)	22.9 (16.7–32.0)	23.0 (16.8–31.5)	22.3 (17.5–30.7)	0.1628
BUN/creatinine ratio§	22.1 (17.5–27.3)	21.9 (16.4–26.9)	21.7 (17.6–26.7)	22.0 (17.4–27.4)	23.1 (18.4–28.7)	0.0052*
ACE‐I or ARBs, *n* (%)	1444 (69.0)	327 (68.2)	393 (70.8)	351 (69.3)	373 (67.8)	0.7148
Loop diuretics† ^,^ ‡ ^,^ §	1617 (77.4)	338 (70.5)	444 (80.2)	393 (77.6)	442 (80.3)	0.0005*
Beta‐blockers	1638 (78.4)	363 (75.7)	450 (81.0)	396 (78.2)	429 (78.1)	0.2267

Continuous variables are presented as median (first–third quartile). Categorical variables are presented as number (percentage). Inter‐subcategory comparisons were performed using analysis of variance or Wilcoxon's signed‐rank test for continuous variables and the chi‐square test for categorical variables. ACE‐I, angiotensin‐converting enzyme inhibitor; ARBs, angiotensin receptor blockers; BP, blood pressure; BUN, blood urea nitrogen; CKD, chronic kidney disease; Cr, creatinine; HR, heart rate; LVEF, left ventricular ejection fraction; WRF, worsening renal function.

† *P* < 0.05 for extreme haemodilution vs. modest haemodilution.

‡ *P* < 0.05 for extreme haemodilution vs. modest haemoconcentration.

§ *P* < 0.05 for extreme haemodilution vs. extreme haemoconcentration.

|| *P* < 0.05 for modest haemodilution vs. modest haemoconcentration.

# *P* < 0.05 for modest haemodilution vs. extreme haemoconcentration.

* *P* < 0.0083 for multiple comparison from Bonferroni correction.

** *P* < 0.05 for modest haemoconcentration vs. extreme haemoconcentration.

Among the laboratory parameters, the haemoglobin concentration at both admission and discharge was similar between the ΔHgb‐based subcategories. Although the BUN/creatinine ratio at discharge was significantly different between the ΔHgb‐based subcategories, there were no differences at admission. The serum creatinine and BUN level at admission and treatment with loop diuretics at discharge were also significantly different between the subcategories.

### Clinical outcomes

Throughout the whole observational period, 452 patients (21.6%) died. Univariate analyses showed that the following parameters were associated with higher all‐cause mortality in the total population: BUN/creatinine ratio at discharge (hazard ratio, 1.016; *P* < 0.0001), age, ischaemic aetiology, systolic blood pressure, resting heart rate, LVEF, and treatment with an ACE‐I or ARB, beta‐blocker, or loop diuretic at discharge (*Table* 2). A higher ΔHgb (i.e. greater haemoconcentration) was also associated with higher post‐discharge mortality (hazard ratio, 0.874; *P* < 0.0001) and showed a trend towards an interaction with the BUN/creatinine at discharge (*P* for interaction = 0.0577) (Supporting Information, *Figure* *S2*). A higher BUN/creatinine ratio at discharge was associated with higher all‐cause mortality in the total population and in the extreme and modest haemodilution and extreme haemoconcentration subcategories, whereas it was not associated with higher mortality in the modest haemoconcentration subcategory [Supporting Information, *Table* *S2*(*A*)–(*E*)].

**Table 2:** Univariate Cox regression analyses for the association of independent variables with post‐discharge all‐cause death

	HR [95% CI]	*P* value
Baseline characteristics		
Age	1.048 [1.039–1.057]	<0.0001*
Men	1.048 [0.866–1.273]	0.6289
CKD	1.814 [1.475–2.248]	<0.0001*
Ischaemic aetiology	1.550 [1.278–1.874]	<0.0001*
Laboratory findings at admission		
Haemoglobin	0.858 [0.822–0.896]	<0.0001*
Creatinine	1.112 [1.055–1.160]	0.0004*
BUN	1.032 [1.027–1.036]	<0.0001*
BUN/creatinine ratio	1.024 [1.016–1.030]	<0.0001*
Vital signs, echocardiographic and laboratory findings, medications at discharge		
Systolic BP	0.991 [0.986–0.997]	0.0028*
Resting HR	1.010 [1.002–1.017]	0.0067*
WRF (ΔCr > 0.3)	1.434 [1.091–1.853]	0.0105*
LVEF	0.990 [0.984–0.996]	0.0020*
Haemoglobin	0.804 [0.768–0.843]	<0.0001*
Creatinine	1.164 [1.086–1.232]	0.0001*
BUN	1.019 [1.016–1.022]	<0.0001*
BUN/creatinine ratio	1.016 [1.009–1.021]	<0.0001*
ACE‐I or ARBs	0.603 [0.500–0.730]	<0.0001*
Loop diuretics	1.455 [1.147–1.871]	0.0017*
Beta‐blockers	0.759 [0.616–0.943]	0.0133*

ACE‐I, angiotensin‐converting enzyme inhibitor; ARBs, angiotensin receptor blockers; BP, blood pressure; BUN, blood urea nitrogen; CI, confidence interval; CKD, chronic kidney disease; Cr, creatinine; HR, hazard ratio; HR, heart rate; LVEF, left ventricular ejection fraction; WRF, worsening renal function.

* *P* < 0.05.

In the multivariate analyses (see Methods section) adjusted for established prognostic factors for ADHF (age, LVEF, and treatment with ACE‐I or ARB, loop diuretics, and beta‐blockers at discharge), a higher BUN/creatinine ratio was independently associated with higher all‐cause mortality in the total population and in the extreme haemodilution and haemoconcentration subcategories (*Table* 3, Model 1). The independent variables that were significantly associated with all‐cause mortality in the univariate analyses were then added for adjustment, which also demonstrated that a higher BUN/creatinine ratio was independently associated with higher all‐cause mortality in the same manner as in Model 1 (*Table* 3, Model 2).

**Table 3:** Multivariate Cox proportional regression analyses of predictive value of the BUN/Cr ratio on all‐cause mortality in the total acute decompensated heart failure population and the subcategories divided according to degree of ΔHgb

Hazard ratio of BUN/Cr ratio on all‐cause mortality in multivariate analysis										
	Total		Extreme haemodilution		Modest haemodilution		Modest haemoconcentration		Extreme haemoconcentration	
	HR [95% CI]	*P* value	HR [95% CI]	*P* value	HR [95% CI]	*P* value	HR [95% CI]	*P* value	HR [95% CI]	*P* value
Model 1	1.018 [1.006–1.023]	0.0018*	1.021 [1.003–1.039]	0.0224*	1.015 [0.994–1.034]	0.1509	NA [NA]	NA	1.021 [1.007–1.032]	0.0067*
Model 2	1.015 [1.005–1.023]	0.0023*	1.024 [1.004–1.042]	0.0140*	1.016 [0.994–1.036]	0.1323	NA [NA]	NA	1.021 [1.007–1.032]	0.0067*

In Model 1, in addition to the BUN/Cr ratio at discharge, we included established prognostic factors for acute decompensated heart failure including age, left ventricular ejection fraction at discharge, and treatment with angiotensin‐converting enzyme inhibitors or angiotensin receptor blockers, loop diuretics, and beta‐blockers at discharge. In Model 2, the independent variables that were significantly associated with all‐cause mortality in the univariate analyses, such as the presence of an ischaemic aetiology, systolic blood pressure, and resting heart rate, were included in the model, in addition to the aforementioned established predictive factors. BUN, blood urea nitrogen; CI, confidence interval; Cr, creatinine; HR, hazard ratio; NA, not available.

* *P* < 0.05.

**Figure 2:**
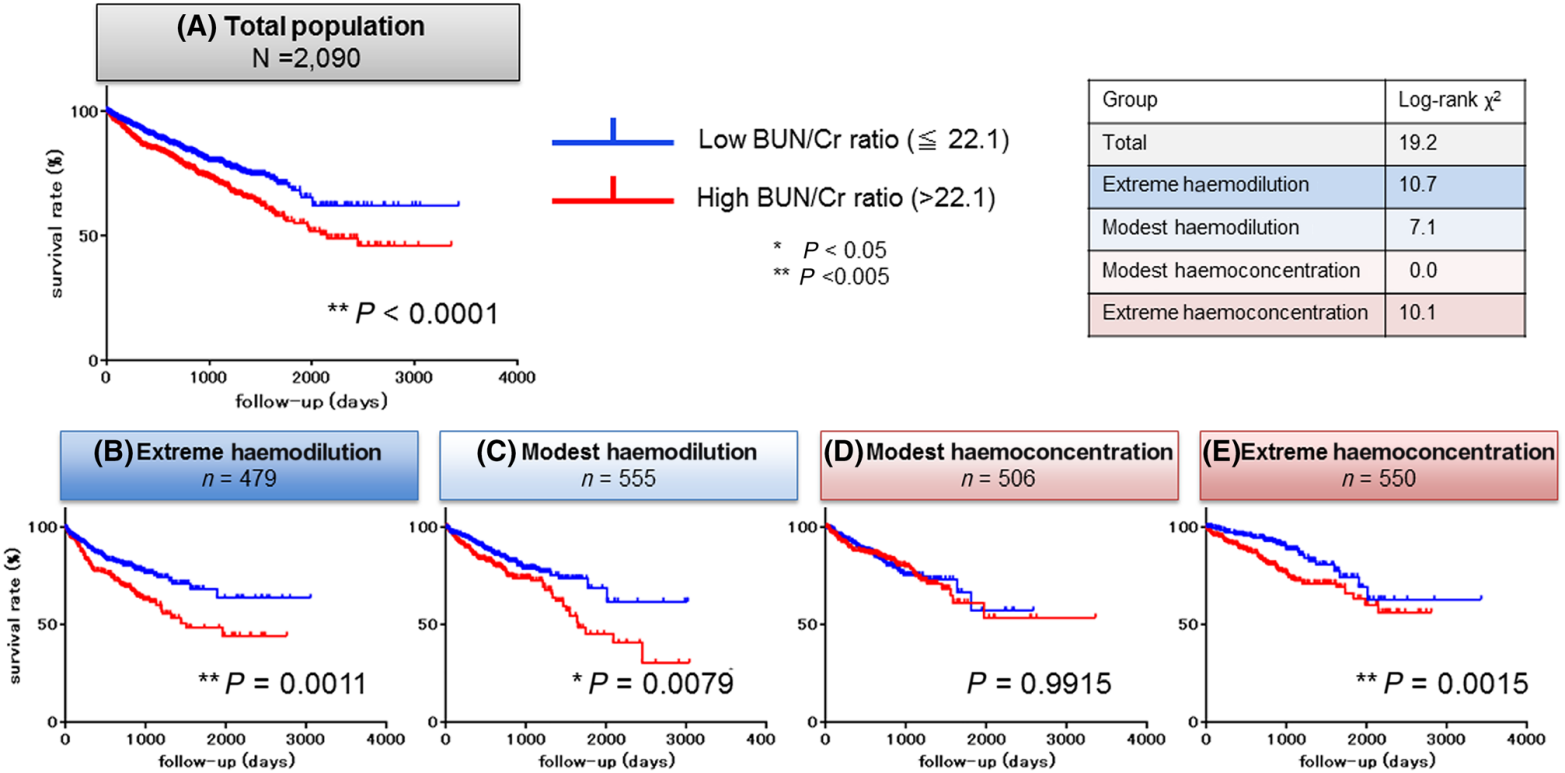
Kaplan–Meier analyses of the blood urea nitrogen (BUN)/creatinine (Cr) ratio at discharge for post‐discharge all‐cause mortality. The median survival times were as follows: total, 771.0 days; extreme haemodilution, 740.0 days; modest haemodilution, 745.0 days; modest haemoconcentration, 764.0 days; extreme haemoconcentration, 819.0 days.

Kaplan–Meier survival analyses showed significantly higher all‐cause mortality in patients with a high BUN/creatinine ratio at discharge in the total population (log‐rank test, *P* < 0.0001) (*Figure* *2* *A*). In the extreme haemodilution, modest haemodilution, and extreme haemoconcentration subcategories, the mortality in the high BUN/creatinine group was significantly higher, whereas in the modest haemoconcentration subcategory, the mortality between the high and low BUN/creatinine ratio groups was comparable [*Figure* *2*(*B*)*–*(*E*)].

## Discussion

The main findings of the present study were (i) that a higher BUN/creatinine ratio at discharge was independently associated with higher post‐discharge all‐cause mortality in patients with ADHF and (ii) that the impact of the high BUN/creatinine ratio at discharge on post‐discharge all‐cause mortality was dependent on the degree of haemoconcentration. By contrast, in the modest haemoconcentration/haemodilution subcategories, a high BUN/creatinine ratio at discharge was not significantly associated with post‐discharge all‐cause mortality (based on multivariate analysis). These findings suggest that the BUN/creatinine ratio at discharge has an important predictive value and that its specific clinical impacts are dependent on the responsiveness to decongestion therapy. We suggest that the clinical implications of a high BUN/creatinine ratio after diuretic therapy may differ and include optimal fluid removal (predominantly observed in modest haemodilution/haemoconcentration subcategories), diuretic therapy‐resistant congestive cardio‐renal syndrome (in the extreme haemodilution subcategory), and excessive fluid removal therapy in poor renal function reserve (in the extreme haemoconcentration subcategory).

### Role of the blood urea nitrogen/creatinine ratio in acute decompensated heart failure

Both serum BUN and creatinine are well‐recognized renal markers^17^ and are associated with outcomes in patients with ADHF.^18^ Because of the different behaviours of BUN and creatinine in the renal tubules, the BUN/creatinine ratio reflects neurohormonal activity in patients with ADHF, and a high BUN/creatinine ratio at admission was proposed as a predictor of acute kidney injury,^4^ cardiovascular rehospitalization, all‐cause death,^3^ and interestingly, HF‐induced reversible renal dysfunction.^2^ In the present study, we also demonstrated a significant association of a higher BUN/creatinine ratio at discharge with higher post‐discharge all‐cause mortality in patients with ADHF. The BUN/creatinine ratio at discharge may reflect not only prerenal or parenchymal renal dysfunction and sympathetic and neurohormonal overactivity but also the responsiveness to in‐hospital decongestion therapy. As such, it may provide incremental predictive value compared with that collected at admission. Note that we divided patients into the two groups according to the median BUN/creatinine ratio value at discharge. The threshold at 22.1 mg/dL was derived from the median of all patients and was similar to the threshold used in a previous study.^4^

### Haemoconcentration as a marker for decongestion therapy

The primary therapeutic objective in most patients with ADHF is optimization of fluid volumes.^19^ Some parameters, such as urine output, plasma B‐type natriuretic peptide, renal function, and body weight, were suggested to be potential metrics for guiding optimized volume removal in patients with HF.^6^ However, there is limited evidence regarding the use of these parameters in hospitalized patients with ADHF. Haemoconcentration was recently proposed as a measure to help guide the correct volume status.^6^, ^19^ Haemoconcentration, defined as an increase in the haemoglobin concentration or haematocrit, was reported to be associated with a lower risk of rehospitalization,^20^, ^21^, ^22^ although there are contrasting findings.^23^ We suggest that by assessing the degree of haemoconcentration rather than its presence, then haemoconcentration is a useful guide for successful fluid removal, while avoiding unfavourable pathologies secondary to excessive decongestion therapy.

### Haemoconcentration‐dependent predictive value of the blood urea nitrogen/creatinine ratio at discharge

In the present study, a higher BUN/creatinine ratio at discharge was significantly associated with post‐discharge all‐cause mortality in all patients with AHDF, although this effect differed between the four ΔHgb‐based subcategories. The impact of a high BUN/creatinine ratio at discharge was strongest in the extreme haemoconcentration subcategory, followed by the extreme haemodilution subcategory, but was not observed in the modest haemoconcentration and haemodilution subcategories. These findings suggest that a high BUN/creatinine ratio at discharge may be an unfavourable clinical phenotype in general ADHF. By contrast, in specific patients whose response to decongestion therapy is limited to the modest range (i.e. modest haemoconcentration and haemodilution), a high BUN/creatinine ratio at discharge may not indicate a poor prognosis. Interestingly, in the extreme haemoconcentration subcategory, the significance of a high BUN/creatinine ratio decreased over time (*Figure* *2* *E*; the Kaplan–Meier curves approach each other by approximately 1000 days after discharge), while in the extreme and moderate haemodilution subcategories, the significance of the high BUN/creatinine ratio became more prominent over time [*Figure* *2*(*B*) and (*C*)]. We speculate that in most patients with extreme haemoconcentration induced by aggressive decongestion therapy, a high BUN/creatinine ratio at discharge suggests reversible rather than permanent renal dysfunction. Thus, the unfavourable effect of a high BUN/creatinine ratio is diminished long after discharge. By contrast, in the modest and extreme haemodilution subcategories, decongestive therapy aimed at haemoconcentration failed, possibly because of poor responsiveness to in‐hospital diuretic therapy. We suggest that in patients with haemodilution, a higher BUN/creatinine ratio at discharge may indicate sustained renal dysfunction or diuretic therapy‐resistant neurohormonal hyperactivity, leading to a poor long‐term prognosis. For patients showing haemodilution, alternative decongestive therapies other than loop diuretics should be considered, such as tolvaptan, which reportedly preserves renal function even in patients with a high BUN/creatinine ratio.^24^, ^25^

### Study limitations

This study has several limitations. First, it was a retrospective design. Thus, causality is impossible to demonstrate, and residual confounding factors cannot be excluded. Second, the administration route and dosing of diuretics were determined by the local attending physicians. As such, these data varied and could not be analysed. Third, plasma B‐type natriuretic peptide or N‐terminal pro B‐type natriuretic peptide concentrations were not sufficiently collected at discharge. These parameters reflect the preload condition, which may modify the clinical implications of the BUN/creatinine ratio, especially in patients with haemodilution. Although the number of patients with plasma B‐type natriuretic peptide or N‐terminal pro B‐type natriuretic peptide data was insufficient for analysis in the present study, these parameters may modify our findings [Supporting Information, *Table* *S4*(*A*)–(*D*)].

## Conclusions

In conclusion, a higher BUN/creatinine ratio at discharge was independently associated with a higher post‐discharge all‐cause mortality in patients with ADHF. The predictive value of a high BUN/creatinine ratio at discharge was haemoconcentration dependent, as it was an unfavourable predictor in patients showing excessive haemoconcentration and haemodilution, but not in those showing modest haemoconcentration/haemodilution. These findings may guide the development of a new therapeutic target and index for patients with ADHF.

## Conflict of interest

Dr. Kohsaka received an unrestricted research grant for the Department of Cardiology, Keio University School of Medicine from Bayer Pharmaceutical Co., Ltd. and Daiichi Sankyo Co., Ltd. The other authors have no conflicts of interest to disclose.

## Funding

This study was supported by a Grant‐in‐Aid for Young Scientists [JPSS KAKENHI, 18K15860 (Y.S.)], a Grant‐in‐Aid for Scientific Research [23591062, 26461088 (T.Y.), 17K09526 (T.K.)], a Health Labour Sciences Research Grant [14528506 (S.K.)], the Sakakibara Clinical Research Grant for Promotion of Sciences [2012, 2013, 2014 (T.Y.)], and a grant from the Japan Agency for Medical Research and Development [201439013C (S.K.)].

## Supporting information


**Figure S1.** The clinical implication of a high BUN/creatinine ratio at discharge may differ according to the response to decongestion therapy as indicated by the degree of hemoconcentration.

**Figure S2.** Hazzard ratio of high BUN/creatinine ratio in Hgb based subcategories.

**Table S1A.** Baseline characteristics at admission in the total population and the four hemoglobin concentration from admission to discharge (ΔHgb)‐based subcategories.
**Table S1B.** Baseline characteristics at discharge in the total population and four ΔHgb‐based subcategories.

**Table S2A.** Univariate and multivariate Cox proportional regression analyses for predictive value of the BUN/creatinine ratio on all‐cause mortality in (A) the total acute decompensated heart failure (ADHF) population, (B) the extreme hemodilution category, (C) the modest hemodilution category, (D) the modest hemoconcentration category, (E) the extreme hemoconcentration category.

**Table S3A.** Univariate analysis of plasma B‐type natriuretic peptide (BNP) level at admission.
**Table S3B**. Multivariate analysis including BNP level at admission.
**Table S3C.** Univariate analysis of N‐terminal pro‐brain natriuretic peptide (NT‐proBNP) level at admission.
**Table S3D.** Multivariate analysis including NT‐proBNP level at admission.
