# Supplementary material for: Clinical implications of the blood urea nitrogen/creatinine ratio in heart failure and their association with haemoconcentration
Source: ESC Heart Fail. 2019 Dec 9;6(6):1274–82. doi: 10.1002/ehf2.12531 (PMC6989280; doi:10.1002/ehf2.12531)

Figure S1. The clinical implication of a high BUN/creatinine ratio at discharge may differ according to the response to decongestion therapy as indicated by the degree of hemoconcentration.

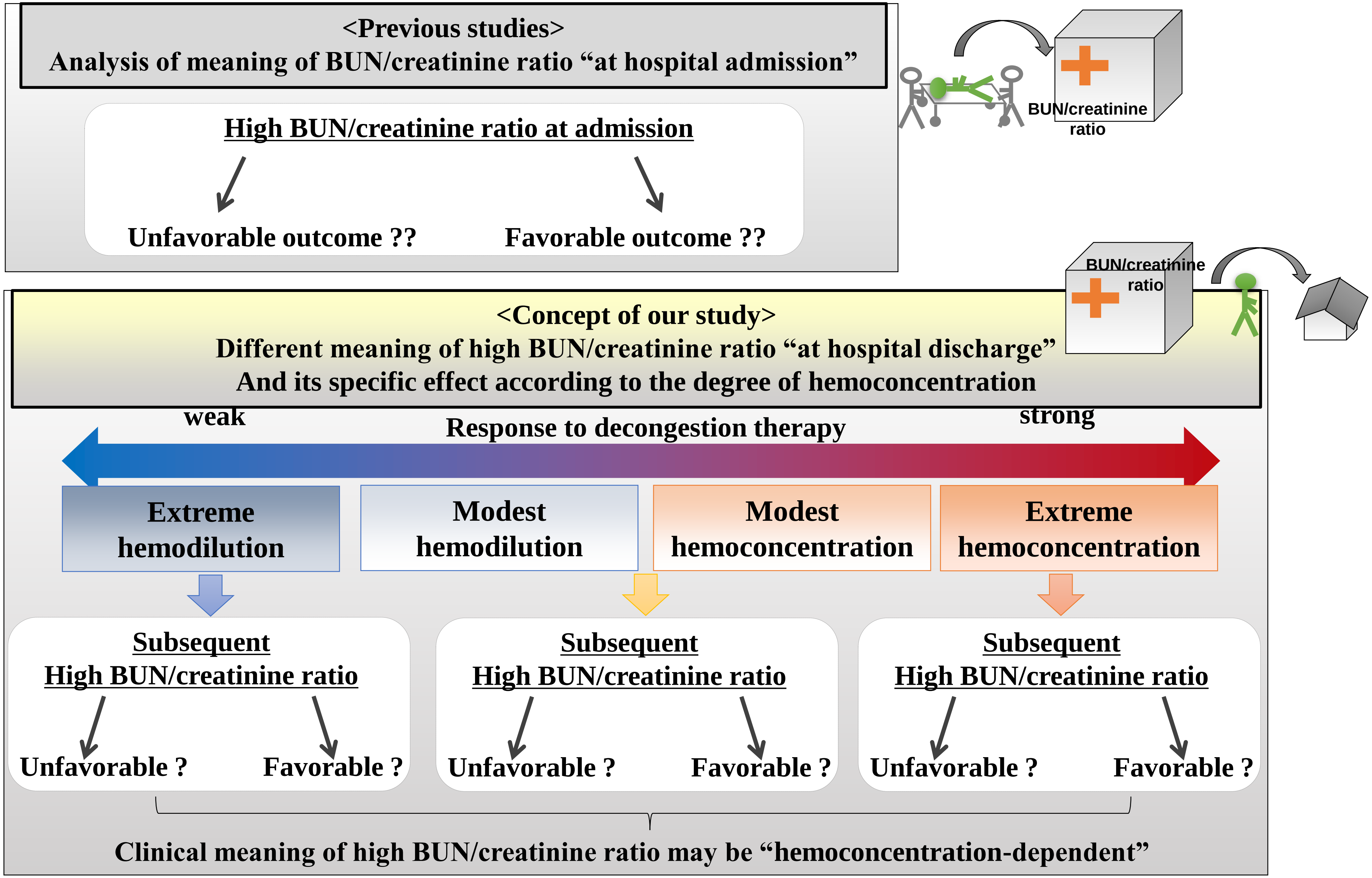

Supplement: Supplementary file 1 — Figure S1. The clinical implication of a high BUN/creatinine ratio at discharge may differ according to the response to decongestion therapy as indicated by the degree of hemoconcentration. [file EHF2-6-1274-s001.pdf]
