# Supplementary figures and images for: Clinical implications of the blood urea nitrogen/creatinine ratio in heart failure and their association with haemoconcentration
Source: ESC Heart Fail. 2019 Dec 9;6(6):1274–82. doi: 10.1002/ehf2.12531 (PMC6989280; doi:10.1002/ehf2.12531)

**Figure S2. Hazzard ratio of high BUN/creatinine ratio in Hgb based subcategories**

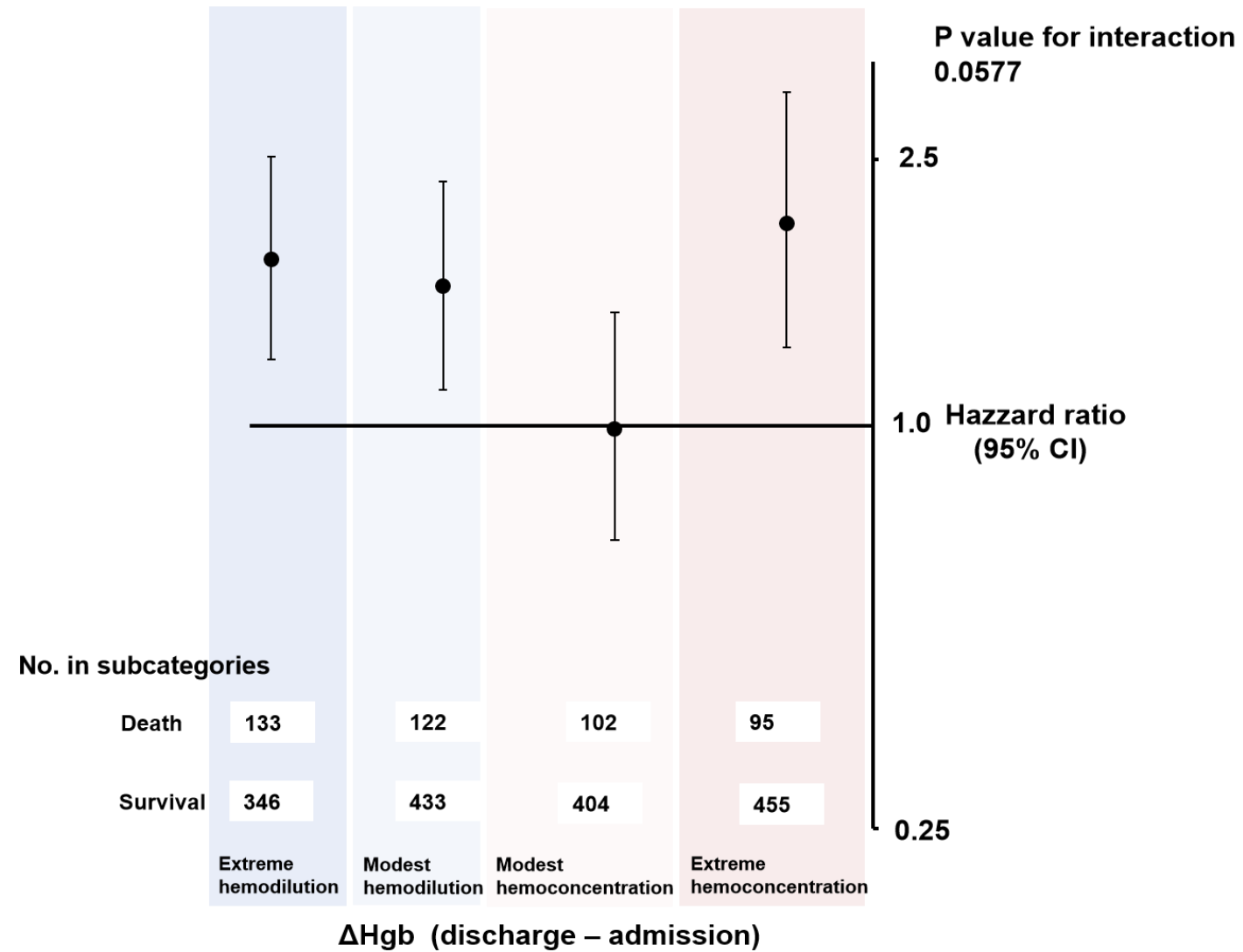

Supplement: Supplementary file 2 — Figure S2. Hazzard ratio of high BUN/creatinine ratio in Hgb based subcategories. [file EHF2-6-1274-s002.pdf]
