# Supplementary material for: Clinical implications of the blood urea nitrogen/creatinine ratio in heart failure and their association with haemoconcentration
Source: ESC Heart Fail. 2019 Dec 9;6(6):1274–82. doi: 10.1002/ehf2.12531 (PMC6989280; doi:10.1002/ehf2.12531)
Supplement: Supplementary file 3 — Table S1A. Baseline characteristics at admission in the total population and the four hemoglobin concentration from admission to discharge (ΔHgb)‐based subcategories. Table S1B. Baseline characteristics at discharge in the total population and four ΔHgb‐based subcategories. [file EHF2-6-1274-s003.pdf]

Table S1A Baseline characteristics at admission in the total population and the four hemoglobin concentration from admission to discharge (ΔHgb)-based subcategories

	Total (n = 2090)	Extreme hemodilution (n = 479)	Modest hemodilution (n = 555)	Modest hemoconcentration (n = 506)	Extreme hemoconcentration (n = 550)	p value (between groups)
**Age, years**[†, ‡, ‖, #]	**76.0 (65.0–83.0)**	**75.0 (63.0–83.0)**	**76.0 (67.0–84.0)**	**77.0 (67.0–83.0)**	**75.0 (63.0–82.0)**	**0.0020***
**Men**	**1294 (61.9)**	**309 (64.5)**	**337 (60.7)**	**308 (60.8)**	**340 (61.8)**	**0.5828**
**SBP, mmHg**[‡, §, ‖, #]	**135 (118–158)**	**138 (116–168)**	**139 (120–162)**	**135 (118–151)**	**132 (115–151)**	**0.0008***
**Resting HR, beats/min**[‡, §]	**90 (72–110)**	**95 (75–118)**	**90 (74–110)**	**87 (70–107)**	**90 (72–109)**	**0.0044***
**CKD**	**1318 (63.0)**	**324 (67.6)**	**357 (64.3)**	**309 (61.0)**	**328 (59.6)**	**0.0389**
**Ischemic etiology**[‡, §, ‖]	**594 (28.4)**	**161 (33.6)**	**173 (31.1)**	**117 (23.1)**	**143 (26.0)**	**0.0007***
**Laboratory findings**						
**Hemoglobin, g/dL**[†, ‡, §, ‖]	**12.4 (10.9–13.9)**	**13.4 (12.1–14.9)**	**12.3 (10.9–13.7)**	**12.2 (10.6–13.5)**	**11.8 (10.4–13.4)**	**<0.0001***
**Creatinine, mg/dL**	**1.00 (0.77–1.30)**	**1.03 (0.80–1.34)**	**1.00 (0.80–1.33)**	**0.96 (0.76–1.30)**	**0.94 (0.73–1.24)**	**0.0040***
**BUN**[§]**, mg/dL**	**20.8 (16.0–28.3)**	**21.9 (16.6–30.2)**	**21.7 (16.4–29.0)**	**20.8 (16.1–27.6)**	**19.3 (14.7–26.5)**	**0.0001***
**BUN/creatinine ratio**	**20.7 (16.8–26.1)**	**20.6 (16.3–27.0)**	**21.0 (17.1–25.8)**	**20.7 (17.0–25.8)**	**20.3 (16.6–26.2)**	**0.5556**
**Sodium, mEq/L**[‡, §]	**140.0 (138.0–142.0)**	**139.6 (137.0–142.0)**	**140.0 (1373.0–142.0)**	**140.0 (138.0–142.0)**	**140.0 (138.0–142.0)**	**0.0002***
**Medications**						
**ACEI or ARBs**	**923 (44.5)**	**205 (42.9)**	**271 (49.3)**	**225 (44.9)**	**222 (40.6)**	**0.0293**
**Digoxin**	**173 (8.3)**	**34 (7.1)**	**43 (7.8)**	**44 (8.7)**	**52 (9.5)**	**0.5278**
**Loop diuretics**	**909 (43.8)**	**204 (42.7)**	**258 (47.1)**	**222 (44.2)**	**225 (41.2)**	**0.2362**

**Beta-blockers**	**914 (44.1)**	**189 (39.8)**	**249 (45.4)**	**234 (46.6)**	**242 (44.4)**	**0.1623**
**Tolvaptan**	**23 (1.4)**	**5 (1.3)**	**6 (1.3)**	**9 (2.2)**	**3 (0.7)**	**0.3540**

Data are presented as mean ± standard deviation or median (1st–3rd quartile) for continuous variables, and as number (percentage) for categorical variables. Inter-subcategory comparisons were performed using analysis of variance or Wilcoxon's signed-rank test for continuous variables, and the chi-square test for categorical variables.

SBP, systolic blood pressure; HR, heart rate; CKD, chronic kidney disease; BUN, blood urea nitrogen; ACE-I, angiotensin-converting enzyme; ARB, angiotensin receptor blocker.

†*P* < 0.05 for extreme hemodilution *vs.* modest hemodilution, ‡*P* < 0.05 for extreme hemodilution *vs.* modest hemoconcentration, §*P* < 0.05 for extreme hemodilution *vs.* extreme hemoconcentration, ||*P* < 0.05 for modest hemodilution *vs.* modest hemoconcentration, #*P* < 0.05 for modest hemodilution *vs.* extreme hemoconcentration, **P* < 0.05 for modest hemoconcentration *vs.* extreme hemoconcentration.

Table S1B Baseline characteristics at discharge in the total population and four ΔHgb-based subcategories

	**Total (n = 2090)**	**Extreme hemodilution (n = 479)**	**Modest hemodilution (n = 555)**	**Modest hemoconcentration (n = 506)**	**Extreme hemoconcentration (n = 550)**	**p value (between groups)**
**Systolic BP, mmHg**	**110 (100–122)**	**110 (100–124)**	**110 (98–122)**	**110 (100–122)**	**110 (100–120)**	**0.4218**
**Resting HR, beats/min**	**70 (61–78)**	**70 (61–80)**	**70 (60–78)**	**70 (62–78)**	**70 (61–80)**	**0.4239**
**WRF (ΔCr > 0.3)**	**211 (10.0)**	**55 (11.4)**	**64 (11.5)**	**43 (8.5)**	**49 (8.9)**	**0.2063**
**LVEF, %**	**44.0 (31.0–58.0)**	**43.0 (30.0–56.0)**	**42.0 (30.0–58.0)**	**45.0 (32.0–58.0)**	**45.0 (32.0–59.0)**	**0.0440**
**Laboratory findings**						
**Hemoglobin, g/dL†, ‡, §, ||, #, ****	**12.4 (10.9–13.9)**	**11.6 (10.5–12.9)**	**12.0 (10.7–13.4)**	**12.5 (11.0–14.0)**	**13.3 (11.9–15.0)**	**<0.0001***
**Creatinine, mg/dL**	**1.10 (0.80–1.30)**	**1.00 (0.76–1.91)**	**1.01 (0.80–1.40)**	**1.00 (0.80–1.30)**	**0.99 (1.80–1.25)**	**0.1735**

**BUN, mg/dL**	**22.4 (16.7–31.0)**	**21.0 (16.0–29.6)**	**22.9 (16.7–32.0)**	**23.0 (16.8–31.5)**	**22.3 (17.5–30.7)**	**0.1628**
**BUN/creatinine ratio§**	**22.1 (17.5–27.3)**	**21.9 (16.4–26.9)**	**21.7 (17.6–26.7)**	**22.0 (17.4–27.4)**	**23.1 (18.4–28.7)**	**0.0052***
**Sodium mEq/L**	**139.0 (137.0–141.0)**	**139.0 (137.0–141.0)**	**139.0 (137.0–141.0)**	**139.2 (137.0–141.0)**	**139.0 (137.0–141.0)**	**0.1213**
**Medications**						
**ACEI or ARBs**	**1,444 (69.0)**	**327 (68.2)**	**393 (70.8)**	**351 (69.3)**	**373 (67.8)**	**0.7148**
**Digoxin‡, §, ‖, #**	**187 (8.9)**	**28 (5.85)**	**34 (6.13)**	**57 (11.2)**	**68 (12.3)**	**<0.0001***
**Loop diuretics†, ‡, §**	**1,617 (77.4)**	**338 (70.)**	**444 (80.2)**	**393 (77.6)**	**442 (80.3)**	**0.0005***
**Beta-blockers**	**1638 (78.4)**	**363 (75.7)**	**450 (81.0)**	**396 (78.2)**	**429 (78.1)**	**0.2267**
**Tolvaptan**	**51 (2.8)**	**12 (2.9)**	**13 (2.7)**	**17 (3.9)**	**9 (1.9)**	**0.3880**

Data are presented as mean ± standard deviation or median (1st–3rd quartile) for continuous variables, and as number (percentage) for categorical variables. Inter-subcategory comparisons were performed using analysis of variance or Wilcoxon's signed-rank test for continuous variables, and the chi-square test for categorical variables.

WRF, worsening renal function; EF, ejection fraction; LVEF, left ventricular ejection fraction.

†$P < 0.05$ for extreme hemodilution *vs.* modest hemodilution, ‡$P < 0.05$ for extreme hemodilution *vs.* modest hemoconcentration, §$P < 0.05$ for extreme hemodilution *vs.* extreme hemoconcentration, ‖$P < 0.05$ for modest hemodilution *vs.* modest hemoconcentration, #$P < 0.05$ for modest hemodilution *vs.* extreme hemoconcentration, *$P < 0.05$ for modest hemoconcentration *vs.* extreme hemoconcentration.
