# Supplementary material for: Clinical implications of the blood urea nitrogen/creatinine ratio in heart failure and their association with haemoconcentration
Source: ESC Heart Fail. 2019 Dec 9;6(6):1274–82. doi: 10.1002/ehf2.12531 (PMC6989280; doi:10.1002/ehf2.12531)
Supplement: Supplementary file 4 — Table S2A. Univariate and multivariate Cox proportional regression analyses for predictive value of the BUN/creatinine ratio on all‐cause mortality in (A) the total acute decompensated heart failure (ADHF) population, (B) the extreme hemodilution category, (C) the modest hemodilution category, (D) the modest hemoconcentration category, (E) the extreme hemoconcentration category. [file EHF2-6-1274-s004.pdf]

Table S2A Univariate and multivariate Cox proportional regression analyses for predictive value of the BUN/creatinine ratio on all-cause mortality in the total acute decompensated heart failure (ADHF) population

	Total population			
	Model 1			
	Univariate analysis		Multivariate analysis	
	HR [95%CI]	p value	HR [95%CI]	p value
BUN/creatinine ratio	1.016 [1.009-1.021]	<0.0001*	1.015 [1.006-1.023]	0.0018*
Age	1.048 [1.039-1.057]	<0.0001*	1.054 [1.045-1.064]	<0.0001*
LVEF	0.990 [0.984-0.996]	0.0020*	0.974 [0.968-0.981]	<0.0001*
Loop diuretics (dis)	1.455 [1.147-1.871]	0.0017*	1.455 [1.038-1.710]	0.0228*
Beta-blockers (dis)	0.759 [0.616-0.943]	0.0133*	0.747 [0.598-0.940]	0.0133*
ACE-I/ARB (dis)	0.603 [0.500-0.730]	<0.0001*	0.631 [0.521-0.766]	<0.0001*
	Model 2			
	Univariate analysis		Multivariate analysis	
	HR [95%CI]	p value	HR [95%CI]	p value
BUN/creatinine ratio	1.016 [1.009-1.021]	<0.0001*	1.015 [1.005-1.023]	0.0023*
Age	1.048 [1.039-1.057]	<0.0001*	1.054 [1.044-1.064]	<0.0001*
ICM	1.550 [1.278-1.874]	<0.0001*	1.300 [1.060-1.590]	0.0188*
Systolic BP, mmHg	0.991 [0.986-0.997]	0.0028*	0.990 [0.984-0.996]	0.0012*
Resting heart rate, beats/min	1.010 [1.002-1.017]	0.0067*	1.010 [1.002-1.017]	0.0068*

**LVEF**	**0.990 [0.984-0.996]**	**0.0020***	**0.978 [0.972-0.985]**	**<0.0001***
**Loop diuretics (dis)**	**1.455 [1.147-1.871]**	**0.0017***	**1.246 [0.974-1.614]**	**0.0402***
**Beta-blockers (dis)**	**0.759 [0.616-0.943]**	**0.0133***	**0.721 [0.577-0.907]**	**0.0057***
**ACE-I/ARB (dis)**	**0.60 [0.50-0.73]**	**<0.0001***	**0.647 [0.532-0.788]**	**<0.0001***

In Model 1, in addition to the BUN/Cr ratio at discharge, we included established prognostic factors for ADHF including age, LVEF at discharge, and treatment with ACE-Is or ARBs, loop diuretics, and beta-blockers at discharge.

In Model 2, the independent variables that were significantly associated with all-cause mortality in the univariate analyses, such as the presence of an ischemic etiology (ICM), SBP, and resting HR, were included the model, in addition to the above-mentioned established predictive factors.

dis, at discharge.

Table S2B Univariate and multivariate Cox proportional regression analyses for predictive value of the BUN/creatinine ratio on all-cause mortality in the extreme hemodilution category

	**Extreme hemodilution**			
	**Model 1**			
	**Univariate analysis**		**Multivariate analysis**	
	**HR [95%CI]**	**p value**	**HR [95%CI]**	**p value**
**BUN/creatinine ratio**	**1.029 [1.011-1.046]**	**0.0015***	**1.021 [1.003-1.039]**	**0.0224***
**Age**	**1.040 [1.025-1.056]**	**<0.0001***	**1.037 [1.022-1.053]**	**<0.0001***
**LVEF**	**0.993 [0.982-1.004]**	**0.2614**		
**Loop diuretics (dis)**	**1.575 [1.053-2.436]**	**0.0260***	**1.622 [1.080-2.515]**	**0.0187***
**Beta-blockers (dis)**	**0.682 [0.474-0.998]**	**0.0494***	**0.852 [0.588-1.257]**	**0.4150**
**ACE-I/ARB (dis)**	**0.650 [0.460-0.928]**	**0.0182***	**0.676 [0.476-0.970]**	**0.0338***
	**Model 2**			
	**Univariate analysis**		**Multivariate analysis**	
	**HR [95%CI]**	**p value**	**HR [95%CI]**	**p value**
**BUN/creatinine ratio**	**1.029 [1.011-1.046]**	**0.0015***	**1024 [1.004-1.042]**	**0.0140***
**Age**	**1.040 [1.025-1.056]**	**<0.0001***	**1.035 [1.020-1.051]**	**<0.0001***
**Ischemic etiology**	**1.604 [1.128-2.265]**	**0.0088***	**1.649 [1.130-2.394]**	**0.0096***
**Systolic BP, mm Hg**	**0.987 [0.978-0.996]**	**0.0058***	**0.985 [0.975-0.995]**	**0.0040***
**Resting heart rate, beats/min**	**1.007 [0.994-1.020]**	**0.2598**		

**LVEF**	**0.993 [0.982-1.004]**	**0.2614**		
**Loop diuretics (dis)**	**1.575 [1.053-2.436]**	**0.0260***	**1.401 [0.928-2.183]**	**0.1103**
**Beta-blockers (dis)**	**0.682 [0.474-0.998]**	**0.0494***	**0.718[0.489-1.072]**	**0.1046**
**ACE-I/ARB (dis)**	**0.650 [0.460-0.928]**	**0.0182***	**0.658 [0.462-0.947]**	**0.0247***

In Model 1, in addition to the BUN/Cr ratio at discharge, we included established prognostic factors for ADHF including age, LVEF at discharge, and treatment with ACE-Is or ARBs, loop diuretics, and beta-blockers at discharge.

In Model 2, the independent variables that were significantly associated with all-cause mortality in the univariate analyses, such as the presence of an ICM, SBP, and resting HR, were included the model, in addition to the above-mentioned established predictive factors.

Table S2C Univariate and multivariate Cox proportional regression analyses for predictive value of the BUN/creatinine ratio on all-cause mortality in the modest hemodilution category

	Modest hemodilution			
	Model 1			
	Univariate analysis		Multivariate analysis	
	HR [95%CI]	p value	HR [95%CI]	p value
BUN/creatinine ratio	1.029 [1.009-1.048]	0.0047*	1.015 [0.994-1.034]	0.1509
Age	1.061 [1.041-1.082]	<0.0001*	1.062 [1.042-1.084]	<0.0001*
LVEF	0.990 [0.978-1.001]	0.0952		
Loop diuretics (dis)	2.324 [1.358-4.338]	0.0014*	2.205 [1.284-4.126]	0.0031*
Beta-blockers (dis)	0.741[0.484-1.177]	0.1973		
ACE-I/ARB (dis)	0.506 [0.350-0.741]	0.0006*	0.585 [0.403-0.858]	0.0065*
	Model 2			
	Univariate analysis		Multivariate analysis	
	HR [95%CI]	p value	HR [95%CI]	p value
BUN/creatinine ratio	1.029 [1.009-1.048]	0.0047*	1.016 [0.994-1.036]	0.1323
Age	1.061 [1.041-1.082]	<0.0001*	1.061 [1.041-1.084]	<0.0001*
Ischemic etiology	1.483 [1.025-2.125]	0.0367*	1.382 [0.939-2.014]	0.0990
Systolic BP, mmHg	0.992 [0.982-1.002]	0.1614		
Resting heart rate, beats/min	1.014 [1.001-1.025]	0.0272*	1.010 [0.997-1.023]	0.1162

**LVEF**	**0.990 [0.978-1.001]**	**0.0952**		
**Loop diuretics (dis)**	**2.324 [1.358-4.338]**	**0.0014***	**2.084 [1.210-3.909]**	**0.0067***
**Beta-blockers (dis)**	**0.741 [0.484-1.177]**	**0.1973**		
**ACE-I/ARB (dis)**	**0.506 [0.350-0.741]**	**0.0006***	**0.589 [0.404-0.868]**	**0.0079***

In Model 1, in addition to the BUN/Cr ratio at discharge, we included established prognostic factors for ADHF including age, LVEF at discharge, and treatment with ACE-Is or ARBs, loop diuretics, and beta-blockers at discharge.

In Model 2, the independent variables that were significantly associated with all-cause mortality in the univariate analyses, such as the presence of an ICM, SBP, and resting HR, were included the model, in addition to the above-mentioned established predictive factors.

Table S2D Univariate and multivariate Cox proportional regression analyses for predictive value of the BUN/creatinine ratio on all-cause mortality in the modest hemoconcentration category

	**Modest hemoconcentration**			
	**Model 1**			
	**Univariate analysis**		**Multivariate analysis**	
	**HR [95%CI]**	**p value**	**HR [95%CI]**	**p value**
**BUN/creatinine ratio**	**1.011 [0.986-1.034]**	**0.3720**		
**Age**	**1.044 [1.024-1.066]**	**<0.0001***	**1.044 [1.024-1.066]**	**<0.0001***
**LVEF**	**0.998 [0.985-1.011]**	**0.8022**		
**Loop diuretics (dis)**	**1.407 [0.872-2.391]**	**0.1662**		
**Beta-blockers (dis)**	**0.828 [0.540-1.309]**	**0.4097**		
**ACE-I/ARB (dis)**	**0.688 [0.463-1.038]**	**0.0744**		
	**Model 2**			
	**Univariate analysis**		**Multivariate analysis**	
	**HR [95%CI]**	**p value**	**HR [95%CI]**	**p value**
**BUN/creatinine ratio**	**1.011 [0.986-1.034]**	**0.3720**		
**Age**	**1.044 [1.024-1.066]**	**<0.0001***	**1.043 [1.023-1.064]**	**<0.0001***
**Ischemic etiology**	**1.630 [1.053-2.465]**	**0.0288***	**1.450 [0.935-2.198]**	**0.0949**
**Systolic BP, mmHg**	**0.998 [0.986-1.010]**	**0.8693**		
**Resting heart rate, beats/min**	**1.005 [0.988-1.022]**	**0.5216**		

**LVEF**	**0.998 [0.985-1.011]**	**0.8022**
**Loop diuretics (dis)**	**1.407 [0.872-2.391]**	**0.1662**
**Beta-blockers (dis)**	**0.828 [0.540-1.309]**	**0.4097**
**ACE-I/ARB (dis)**	**0.688 [0.463-1.038]**	**0.0744**

In Model 1, in addition to the BUN/Cr ratio at discharge, we included established prognostic factors for ADHF including age, LVEF at discharge, and treatment with ACE-Is or ARBs, loop diuretics, and beta-blockers at discharge.

In Model 2, the independent variables that were significantly associated with all-cause mortality in the univariate analyses, such as the presence of an ICM, SBP, and resting HR, were included the model, in addition to the above-mentioned established predictive factors.

Table S2E. Univariate and multivariate Cox proportional regression analyses for predictive value of the BUN/creatinine ratio on all-cause mortality in the extreme hemoconcentration category

	Extreme hemoconcentration			
	Model 1			
	Univariate analysis		Multivariate analysis	
	HR [95%CI]	p value	HR [95%CI]	p value
BUN/creatinine ratio	1.015 [1.004-1.022]	0.0108*	1.021 [1.007-1.032]	0.0067*
Age	1.053 [1.034-1.073]	<0.0001*	1.068 [1.046-1.091]	<0.0001*
LVEF	0.982 [0.968-0.995]	0.0081*	0.960 [0.946-0.974]	<0.0001*
Loop diuretics (dis)	0.998 [0.611-1.722]	0.9964		
Beta-blockers (dis)	0.865 [0.546-1.428]	0.5596		
ACE-I/ARB (dis)	0.528 [0.353-0.793]	0.0023*	0.521 [0.345-0.790]	0.0023*
	Model 2			
	Univariate analysis		Multivariate analysis	
	HR [95%CI]	p value	HR [95%CI]	p value
BUN/creatinine ratio	1.015 [1.004-1.022]	0.0108*	1.021 [1.007-1.032]	0.0067*
Age	1.053 [1.034-1.073]	<0.0001*	1.068 [1.046-1.091]	<0.0001*
Ischemic etiology	1.370 [0.888-2.076]	0.1510		
Systolic BP, mmHg	0.990 [0.977-1.002]	0.1143		

**Resting heart rate, beats/min**	**1.010 [0.993-1.026]**	**0.2276**		
**LVEF**	**0.982 [0.968-0.995]**	**0.0081***	**0.960 [0.946-0.974]**	**<0.0001***
**Loop diuretics (dis)**	**0.998 [0.611-1.722]**	**0.9964**		
**Beta-blockers (dis)**	**0.865 [0.546-1.428]**	**0.5596**		
**ACE-I/ARB (dis)**	**0.528 [0.353-0.793]**	**0.0023***	**0.521 [0.345-0.790]**	**0.0023***

In Model 1, in addition to the BUN/Cr ratio at discharge, we included established prognostic factors for ADHF including age, LVEF at discharge, and treatment with ACE-Is or ARBs, loop diuretics, and beta-blockers at discharge.

In Model 2, the independent variables that were significantly associated with all-cause mortality in the univariate analyses, such as the presence of an ICM, SBP, and resting HR, were included the model, in addition to the above-mentioned established predictive factors.
