# Supplementary material for: Clinical implications of the blood urea nitrogen/creatinine ratio in heart failure and their association with haemoconcentration
Source: ESC Heart Fail. 2019 Dec 9;6(6):1274–82. doi: 10.1002/ehf2.12531 (PMC6989280; doi:10.1002/ehf2.12531)
Supplement: Supplementary file 5 — Table S3A. Univariate analysis of plasma B‐type natriuretic peptide (BNP) level at admission. Table S3B. Multivariate analysis including BNP level at admission. Table S3C. Univariate analysis of N‐terminal pro‐brain natriuretic peptide (NT‐proBNP) level at admission. Table S3D. Multivariate analysis including NT‐proBNP level at admission. [file EHF2-6-1274-s005.pdf]

Table S3A. Univariate analysis of plasma B-type natriuretic peptide (BNP) level at admission

**Hazard ratio of BNP on all-cause mortality in univariate analysis**									
**Total**		ΔHgb-based subcategories							
		**Extreme hemodilution**		**Modest hemodilution**		**Modest Hemoconcentration**		**Extreme hemoconcentration**	
**HR [95%CI]**	**p value**	**HR [95%CI]**	**p value**	**HR [95%CI]**	**p value**	**HR [95%CI]**	**p value**	**HR [95%CI]**	**p value**
**1.000[1.000-1.000]**	**0.0001***	**1.000[1.000-1.000]**	**0.0466***	**1.000[0.999-1.000]**	**0.0698**	**1.000[1.000-1.000]**	**0.0388***	**1.000[0.999-1.000]**	**0.1302**

Note that BNP was collected in 59.9% of cases.

Table S3B Multivariate analysis including BNP level at admission

**Hazard ratio of BUN/Cr ratio and BNP on all-cause mortality in multivariate analysis**											
		**Total**		ΔHgb-based subcategories							
				**Extreme hemodilution**		**Modest hemodilution**		**Modest hemoconcentration**		**Extreme hemoconcentration**	
		**HR [95%CI]**	**p value**	**HR [95%CI]**	**p value**	**HR [95%CI]**	**p value**	**HR [95%CI]**	**p value**	**HR [95%CI]**	**p value**
**Model 1**	**BUN/Cr**	**1.011[1.000-1.020]**	**0.0395***	**1.027[1.004-1.048]**	**0.0184***	**1.015[0.994-1.034]**	**0.1509**	**NA [NA]**	**NA**	**1.021[1.007-1.032]**	**0.0067***
	**BNP**	**1.000 [0.999-1.000]**	**0.0866**	**1.000 [0.999-1.000]**	**0.3948**	**NA [NA]**	**NA**	**1.000 [0.999-**	**0.1528**	**NA [NA]**	**NA**

							1.096]			
**Model 2**	**BUN/Cr**	**1.011[0.999-1.021]**	**0.0605**	**1.031[1.008-1.054]**	**0.0088***| **1.016[0.994-1.036]**	**0.1323**	**NA [NA]**	**NA**	**1.021[1.007-1.032]**	**0.0067***
	**BNP**	**1.000 [0.999-1.000]**	**0.0570**	**1.000 [0.999-1.000]**	**0.6977**	**NA [NA]**	**NA**	**1.000 [0.999-1.001]**	**0.1542**	**NA [NA]**	**NA**

Table S3C Univariate analysis of N-terminal pro-brain natriuretic peptide (NT-proBNP) level at admission

**Hazard ratio of NT-pro BNP on all-cause mortality in univariate analysis**									
**Total**		ΔHgb-based subcategories							
		**Extreme hemodilution**		**Modest hemodilution**		**Modest hemoconcentration**		**Extreme hemoconcentration**	
**HR [95%CI]**	**p value**	**HR [95%CI]**	**p value**	**HR [95%CI]**	**p value**	**HR [95%CI]**	**p value**	**HR [95%CI]**	**p value**
**1.000[1.000-1.000]**	**<0.0001***	**1.000[0.999-1.000]**	**0.3343**	**1.000[1.000-1.000]**	**0.0001***	**1.000[0.999-1.000]**	**0.1059**	**1.000[1.000-1.000]**	**0.0340***

Note that NT-ProBNP was collected in 42.3% of cases.

Table S3D Multivariate analysis including NT-proBNP level at admission

**Hazard ratio of BUN/Cr ratio and NT-proBNP on all-cause mortality in multivariate analysis**		
	**Total**	ΔHgb-based subcategories

				Extreme hemodilution		Modest hemodilution		Modest hemoconcentration		Extreme hemoconcentration	
		HR [95%CI]	p value	HR [95%CI]	p value	HR [95%CI]	p value	HR [95%CI]	p value	HR [95%CI]	p value
Model 1	BUN/Cr	1.021[1.003-1.039]	0.0211*	1.021[1.003-1.039]	0.0224*	1.044[1.011-1.075]	0.0082*	NA [NA]	NA	1.019[0.982-1.052]	0.2985
	NT-proBNP	1.000 [1.000-1.000]	0.0206*	NA [][NA]	NA	1.000 [1.000-1.000]	0.0004*	NA [NA]	NA	1.000 [0.999-1.000]	0.8353
Model 2	BUN/Cr	1.021[1.003-1.039]	0.0227*	1024[1.004-1.042]	0.0140*	1.049[1.016-1.081]	0.0040*	NA [NA]	NA	1.019[0.982-1.052]	0.2985
	NT-proBNP	1.000 [1.000-1.000]	0.0132*	NA [NA]	NA	1.000 [1.000-1.000]	0.0019	NA [NA]	NA	1.000 [0.999-1.000]	0.8353

Independent variables in Model 1: Age, LVEF (at discharge), ACE-Is or ARBs (at discharge), loop diuretics (at discharge), and beta-blockers (at discharge), and BUN/creatinine ratio (at discharge), plus either BNP (at admission) or NT-proBNP (at admission).

Independent variables in Model 2: In addition to variables included in Model 1, ischaemic cardiac disease, systolic blood pressure, and resting heart rate.
